# Transmembrane protease, serine 4 (TMPRSS4) is upregulated in IPF lungs and increases the fibrotic response in bleomycin-induced lung injury

**DOI:** 10.1371/journal.pone.0192963

**Published:** 2018-03-12

**Authors:** Ana Valero-Jiménez, Joaquín Zúñiga, José Cisneros, Carina Becerril, Alfonso Salgado, Marco Checa, Ivette Buendía-Roldán, Criselda Mendoza-Milla, Miguel Gaxiola, Annie Pardo, Moisés Selman

**Affiliations:** 1 Instituto Nacional de Enfermedades Respiratorias, Ismael Cosío Villegas, Ciudad de México, México; 2 Facultad de Ciencias, Universidad Nacional Autónoma de México, Ciudad de México, México; University of Pittsburgh, UNITED STATES

## Abstract

Idiopathic pulmonary fibrosis (IPF) is a chronic and progressive lung disease characterized by epithelial cell activation, expansion of the fibroblast population and excessive extracellular matrix accumulation. The mechanisms are incompletely understood but evidence indicates that the deregulation of several proteases contributes to its pathogenesis. Transmembrane protease serine 4 (TMPRSS4) is a novel type II transmembrane serine protease that may promote migration and facilitate epithelial to mesenchymal transition (EMT), two critical processes in the pathogenesis of IPF. Thus, we hypothesized that over-expression of TMPRSS4 in the lung could promote the initiation and/or progression of IPF. In this study we first evaluated the expression and localization of TMPRSS4 in IPF lungs by real time PCR, western blot and immunohistochemistry. Then we examined the lung fibrotic response in wild-type and TMPRSS4 deficient mice using the bleomycin-induced lung injury model. We found that this protease is upregulated in IPF lungs, where was primarily expressed by epithelial and mast cells. Paralleling the findings in vivo, TMPRSS4 was expressed by alveolar and bronchial epithelial cells in vitro and unexpectedly, provoked an increase of E-cadherin. No expression was observed in normal human or IPF lung fibroblasts. The lung fibrotic response evaluated at 28 days after bleomycin injury was markedly attenuated in the haplodeficient and deficient TMPRSS4 mice. By morphology, a significant reduction of the fibrotic index was observed in KO and heterozygous mice which was confirmed by measurement of collagen content (hydroxyproline: WT: 164±21.1 μg/lung versus TMPRSS4 haploinsufficient: 110.2±14.3 μg/lung and TMPRSS4 deficient mice: 114.1±24.2 μg/lung (p<0.01). As in IPF, TMPRSS4 was also expressed in epithelial and mast cells. These findings indicate that TMPRSS4 is upregulated in IPF lungs and that may have a profibrotic role.

## Introduction

Idiopathic pulmonary fibrosis (IPF) is the most aggressive of the interstitial lung diseases (ILD), leading to respiratory failure and premature death with a median life expectancy of about three years from diagnosis [1, 2]. IPF is characterized by the abnormal activation of alveolar epithelial cells, which produce a variety of mediators causing migration, proliferation and differentiation of fibroblasts into myofibroblasts which in turn secrete large amounts of extracellular matrix resulting in abnormal remodeling of the lung architecture [1, 3, 4].

A number of mediators including cytokines, growth factors and proteolytic enzymes have been implicated in the development of the disease. However, the biopathological processes leading to IPF and the mechanisms responsible for the abnormal activation of epithelial cells and fibroblasts have not been elucidated.

Proteases play crucial roles in a large variety of physiologic and pathological processes including the degradation of basement membrane and extracellular matrix (ECM) and tissue remodeling [5, 6], and particularly, several matrix metalloproteases have been found deregulated in IPF [7]. In humans, more than 2% of the genes code for a complex system of more than 700 proteases and inhibitors of proteases [8].

Recently, much attention has been focused on the role of type II transmembrane serine proteases (TTSPs) in physiological and pathological processes. These proteases are cell surface-associated enzymes that have in common an extracellular domain, a single-pass transmembrane domain, a short intracellular domain, and a variable-length stem region containing modular structural domains [9–11]. Although a few of the TTSPs are expressed across several tissues and cell types, in general these enzymes demonstrate relatively restricted expression patterns, indicating that they may have tissue-specific functions [9].

To date, 20 TTSPs have been identified in mouse and humans and most of them are over-expressed in a variety of tumor or other pathologic conditions compared to normal tissues. In addition to cancer, altered expression and activity of these enzymes has been found in some chronic non-neoplastic disorders such as atherosclerosis, arthritis and neurodegenerative processes, where may contribute to the initiation or progression of these diseases [7].

TMPRSS4 is a novel TTSP that is highly expressed in a variety of cancers where it has been implicated in their pathogenesis and invasion [12–15]. Moreover, a recent meta-analysis study indicates that high TMPRSS4 expression in solid tumor tissues is associated with poor overall survival and short time to tumor progression [16].

To date this protease has not been studied in fibrotic lung diseases. Since TMPRSS4 is involved in some processes that may contribute to the pathogenesis of IPF such as migration and epithelial to mesenchymal transition (EMT) we hypothesized that it may be involved in the initiation and/or progression of this disease. Our results demonstrated that TMPRSS4 is upregulated in epithelial and mast cells in IPF lungs and that the fibrotic response to bleomycin is attenuated in TMPRSS4-deficient mice suggesting a profibrotic role for this protease.

## Material and methods

### Quantitative PCR

TMPRSS4 gene expression was quantified in lung homogenates from patients with IPF and hypersensitivity pneumonitis (HP) while normal control RNA samples were obtained from commercial sources (Ambion AM7968, Agilent 540019). For some experiments we used RNA from different cell cultures. Total RNA was extracted using TRIzolreagent (Invitrogen Life Technologies, Grand Island, NY). Total RNA (1 μg) was reverse-transcribed using a high-capacity cDNA reverse transcription kit (Thermo Scientific, K1632) according to the manufacturer’s instructions. TaqMan probes were designed at Applied Biosystems (4331182) and TaqMan PCR Master Mix (4304437) and qPCR was performed in a Rotor-Gene Q Instrument (QIAGEN, Hilden, Germany). Samples were run in triplicates using 18S rRNAas reference gene.

### Cell culture

Human alveolar (A549) (CCL-185) and bronchial (HBE4-E6/E7) (CRL-2078) epithelial cell lines, lung epithelial cells from rat (RLE-6TN; CRL-2300) and human lung fibroblasts CCD-25Lu (CCL-215) and CCD-8Lu (CCL-201) were obtained from American Type Culture Collection (ATCC). Cells were grown at 37°C in a gas mixture of 5% CO_2_ / 95% air in 25 cm^2^ culture flasks (T-25; Corning, Costar) containing culture medium supplemented with 10% fetal bovine serum (FBS) (GIBCO Laboratories, Grand Island, NY), 100 U/ml of penicillin, 100 μg/ml of streptomycin, and 2.5 mg/ml of amphotericin B until were plated for experiments.

A549 cells and fibroblasts were grown in F12 medium (GIBCO, 21700–075) and bronchial cells in Defined Keratinocyte-SFM (GIBCO, 10744) supplemented with bovine pituitary extract (0.05 mg/mL), epidermal growth factor (5 ng/mL) and cholera toxin (10 ng/mL). RLE-6TN cells were grown in Ham´s F12 medium supplemented with bovine pituitary extract (0.01 mg/mL), insulin (5 ng/mL), insulin-like growth factor (2.5 ng/mL), transferrin (1.25 ug/mL), EGF (2.5 ng/mL), and 10% FBS.

### Growth rate assay

Epithelial cells were seeded in 96-well culture plates at a cell density of 2800 per well and incubated in F12 supplemented with 10% FBS at standard conditions. After 24 h, the medium was replaced by serum-free medium alone or with human recombinant TMPRSS4 [Origene, TP303972 (0.1, 1, 10 and 100 ng/mL)], and the cells were maintained in culture for 48 or 96 h. Cell growth was determined using the cell proliferation reagent WST-1 (Boehringer, Mannheim, Germany) as described [17]. All assays were performed in triplicate.

### Immunohistochemistry (IHC)

We examined the localization of TMPRSS4 in five IPF and three normal lungs. Immunohistochemical analysis was performed as described [17]. Briefly, formalin-fixed, paraffin-embedded lung tissues were obtained from biopsy or autopsy specimens of individuals with IPF and controls in compliance with institutional review board-approved protocols. Lung tissue blocks were cut in 3 μm sections. After antigen unmasking using citrate buffer at 90°C for 30 min, and blocking with 2% of normal pig serum in PBS, intervening sections were incubated for 18 h at 4°C with anti-TMPRSS4 monoclonal antibody [Santa Cruz Biotechnology/ sc-376415 (1:100)] diluted in PBS with 2% of serum. Sections were then incubated with a secondary biotinylated anti-immunoglobulin antibody followed by horseradish peroxidase-conjugated streptavidin (BioGenex, San Ramon, CA). 3-Amino-9-etilcarbazol in acetate buffer containing 0.05% H_2_O_2_ was used as substrate. The primary antibody was replaced by nonimmune serum for negative control slides [17]. The same protocol was used for mouse lung sections from the bleomycin-induced lung fibrosis model but using anti-TMPRSS4 polyclonal antibody [Cusabio/ CSB-PA023926GA01Hu (1:50)].

### Immunofluorescence staining and confocal microscopy

For immunofluorescence, 3 μm thick tissue sections adhered to silanized slides (Sigma-Aldrich, St. Louis MO, USA) were used. After heat-induced antigen retrieval in citrate buffer, samples were permeabilized and blocked in a single step incubating them for 30 minutes in PBS with 2% normal goat/porcine serum and 0.5% Triton X-100 (Research Organic, Cleveland, OH, USA) at room temperature (RT). Afterwards samples were washed and incubated at 4ºC overnight with primary antibodies: anti-TMPRSS4 [Santa Cruz Biotechnology/ sc-376415 (1:100)], anti-Tryptase [Abcam/ ab-151757 (1:250)] and anti-Chymase [Abcam/ ab-111239 (1:300)]. The next day samples were washed and incubated for 1 hour at RT with fluorescent secondary antibodies (Alexa Fluor[AF]-488 conjugated donkey anti-Rabbit IgG, Dylight-549 conjugated donkey anti-mouse IgG and AF-647 conjugated donkey anti-goat IgG; (Jackson Immunoresearch, West Grove, PA). Nuclei were stained with NucBlue (Life Technologies, Carlsbad, CA). Samples were mounted with ProLong Gold mounting media (Life Technologies). All experiments included controls with/without primary antibody or with/without secondary antibody. Imaging was performed with an FV-1000 confocal laser scanning microscope (Olympus Corporation, Tokyo, Japan) in sequential scanning mode to image each fluorochrome separately.

### Western blot

Cellular lysates from alveolar (A549) and bronchial (HBE4-E6/E7) (CRL-2078) human epithelial cells, and from human normal and IPF fibroblasts were prepared using RIPA buffer (50 mMTris·HCl, pH 8, 150 mMNaCl, 1% Nonidet P-40, 0.1% SDS, 1% Triton X-100, 1 mMNaF, 1 mM Na3VO4, 1 mM phenylmethylsulfonyl fluoride, 1 μg/ml leupeptin, 1 μg/ml aprotinin). Protein concentration was quantified using Bradford assay (Bio-Rad, Hercules CA). Proteins were mixed with Laemmli sample buffer and 40 μg were loaded on denaturing 10% SDS-polyacrylamide gels, electro-transferred to nitrocellulose membranes and subsequently blocked with 5% skim milk for 1 h. Membranes were incubated overnight at 4°C or 1 h at room temperature with the primary antibodies: TMPRSS4 [Santa Cruz Biotechnology/ sc-376415 (1:500)], E-cadherin [BioGenex/ MU390-UC (1:150); Santa Cruz Botechnology/ sc-7870 (1:200)] and alpha-SMA [SIGMA/ A2547 (1:100)]. In some experiments, immunoblotting was performed in A549 cells stimulated with 100 ng/mL TMPRSS4 recombinant protein (Origene/ TP303972) and 5 ng/mLTGF-β1 (R&D Systems/ 240-B/CF). After primary antibodies incubation, the membranes were incubated with horseradish peroxidase-conjugated anti-mouse IgG or anti-rabbit IgG antibodies (Invitrogen Life Technologies) for 1 h at room temperature. Signal was detected by chemiluminescence (SuperSignal West Pico Chemiluminescent Substrate, Thermo Scientific / 34080) and densitometry was performed using the Image Lab software (Bio-Rad Laboratories, Inc.). All samples were normalized against beta-tubulin [Santa Cruz Biotechnology/ sc-5274HRP (1:200)] or beta-actin [Santa Cruz Biotechnology/ sc-47778 (1:200)].

### Bleomycin-induced lung fibrosis

#### Ethics approval and consent to participate

The study protocol for tissue donation from patients and all experiments, including those on mice, were conducting according to the institutional guidelines. Informed consent was obtained for each individual patient. Animal use had complied with the Animal Care guidelines. This protocol was approved by the Bioethics Committee of the Instituto Nacional de Enfermedades Respiratorias Ismael Cosío Villegas (INER) (B30-12) in Mexico City.

#### Animals

TMPRSS4 deficient mice with C57BL/6 background were obtained from the MMRRC at University of California, Davis. Wild-type littermates were used as controls. Mice were housed in specific pathogen-free facilities and use for experiments at 8–12 weeks of age. Animals received sterile food and water *ad libitum* and were handled aseptically. Genotyping of the mice was performed by direct polymerase chain reaction of lysed murine tissue (PRIMERS: for: AACTTCACAGAAGCACTGGCC, rev: TGGGATTCAAACGTGGTTCTT and for: GCAGCGCATCGCCTTCTATTC, rev: TGGGATTCAAACGTGGTCCTG).

#### Anesthesia

Mice were anesthetized with AVERTIN, a mixture of 222-tribromoethanol (SIGMA Aldrich, T4840-2) and t-amyl alcohol (SIGMA Aldrich, A1685) for the instillation of BLM and also to euthanize them at the end of experiments which was done by Exsanguination/Cardiac Perfusion. We made all the efforts to alleviate suffering.

#### Bleomycin-induced pulmonary fibrosis

We first made a dose-response analysis (1, 3, 5 and 7 U/Kg), and found that the best model was obtained with 7U/Kg (0.07 U/10g) with a similar 15–20% mortality between WT, HT and KO mice. Wild type (WT), heterozygous (HT) and deficient (KO) mice were randomly divided into two groups and instilled with PBS (control group) or bleomycin (experimental group). BLM sulfate (BLEOLEM) was dissolved in PBS and instilled intratracheally at a dose of 7 U/Kg (final volume: 50 μl/mouse) using a single application. On day 28, mice were sacrificed and lungs were removed. Left lungs were used to measure hydroxyproline content and right lungs were fixed in 10% formaldehyde, embedded in paraffin, and sectioned. Tissue sections of 3 μm thickness were stained with hematoxylin-eosin (HE) and Masson’s Trichrome. The severity of lung fibrosis was determined using a semiquantitative histopathological scoring method. First, it was determined the extent of lesions ranging from 0–100% of the slide and then the percent of fibrosis by trichromic staining. A fibrotic index was calculated multiplying the extent of the lesions X the percent of fibrosis [18].

#### Hydroxyproline assay

Collagen quantification was performed by hydroxyproline assay as previously described [19]. Left lungs were consistently used to allow comparisons. Data were expressed as micrograms of hydroxyproline per lung.

### Statistical analysis

Differences were evaluated by unpaired T-test (parametric data) with Welch´s correction for real-time PCR in tissues. For the other experiments P values were calculated by using one-way ANOVA test. All data were expressed as mean ± SD of 3 or 4 determinations. P values <0.05 were considered statistically significant.

## Results

### TMPRSS4 is overexpressed in IPF lungs

**Fig 1:**
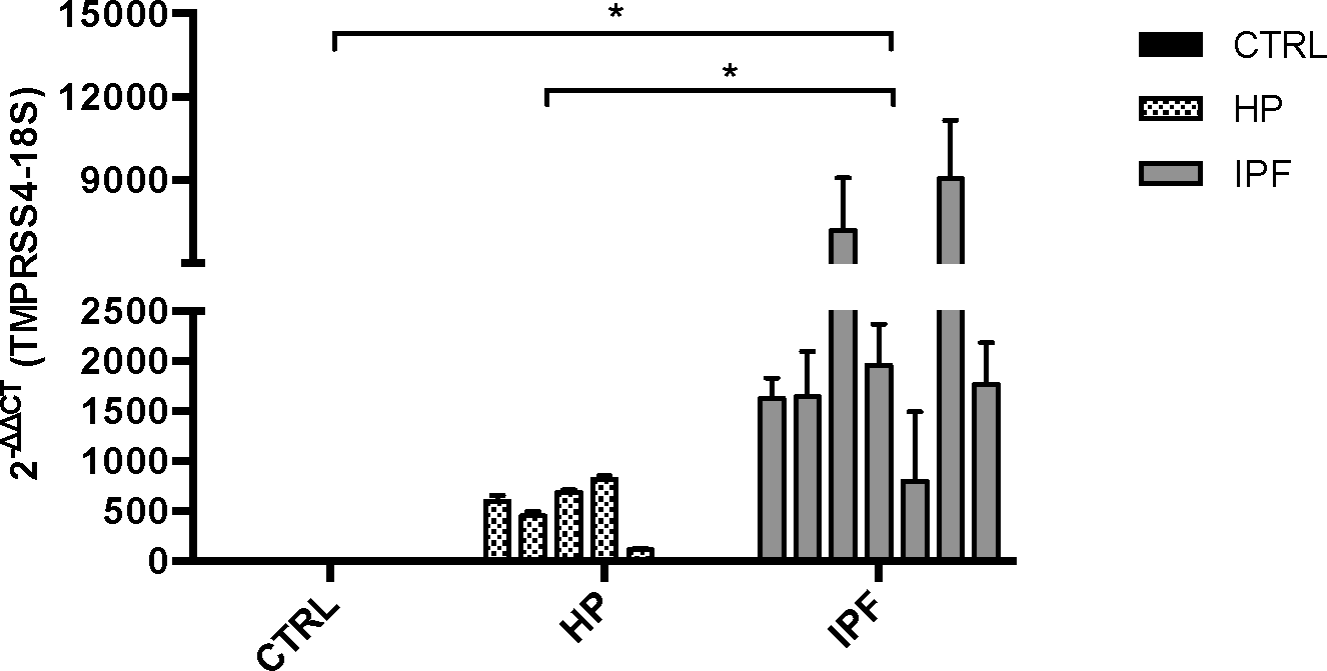
TMPRSS4 is upregulated in idiopathic pulmonary fibrosis (IPF) lungs. Gene expression of TMPRSS4 was quantified by real-time PCR in total RNA obtained from IPF (n = 7), hypersensitivity pneumonitis (HP) (n = 6) and normal lungs (n = 4). Strong upregulation was observed in IPF tissues compared with HP and control lungs. Data are expressed as means ± SD of copy number normalized to 18S rRNA; * p<0.05, IPF versus control and HP.

We first evaluated by real-time qPCR the expression of TMPRSS4 in lung samples from IPF patients (n = 7) compared with normal tissues (n = 4) and hypersensitivity pneumonitis (HP, n = 6). Demographic and functional data of the patients are included in **S1 Table**. HP samples were used because it represents an inflammatory-driven ILD that often evolve to fibrosis. As shown in **Fig 1** TMPRSS4 was virtually not expressed in control lungs while it was modestly expressed in HP and strongly upregulated in IPF tissues (p<0.05, IPF versus controls and HP).

### TMPRSS4 is expressed by epithelial and mast cells

**Fig 2:**
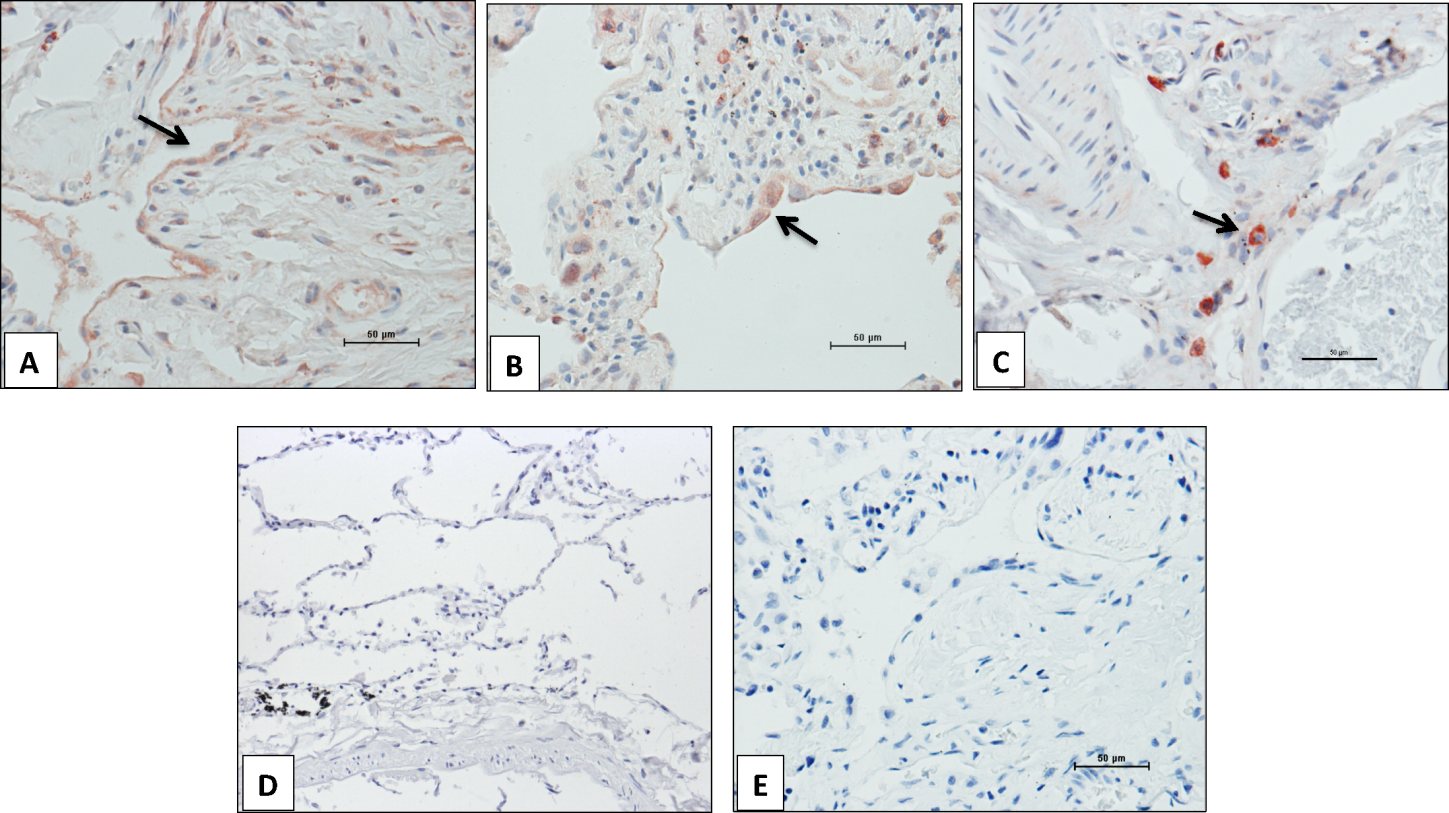
Immunolocalization of TMPRSS4 in IPF and control lungs. Representative photomicrographs of immunohistochemical staining performed with antibody against TMPRSS4 in lung tissue sections. Strong staining was observed in epithelial (panels A and B) and interstitial cells (panel C) in IPF lungs (n = 5), whereas no positive labeling was detected in normal lungs (n = 3) (panel D). Panel E shows the negative control where the specific antibody was omitted. All sections were counterstained with hematoxylin. Arrows indicate positive cells.

TMPRSS4 cell localization was examined by immunohistochemistry. Paralleling the gene expression results, no immunoreactive protein was observed in the control lungs. In IPF lungs, strong positive labeling was noticed in interstitial cells while a positive but weaker staining was observed in alveolar epithelial cells (**Fig 2**). The expression of TMPRSS4 was observed primarily in the fibrotic areas. No staining was detected in fibroblasts.

**Fig 3:**
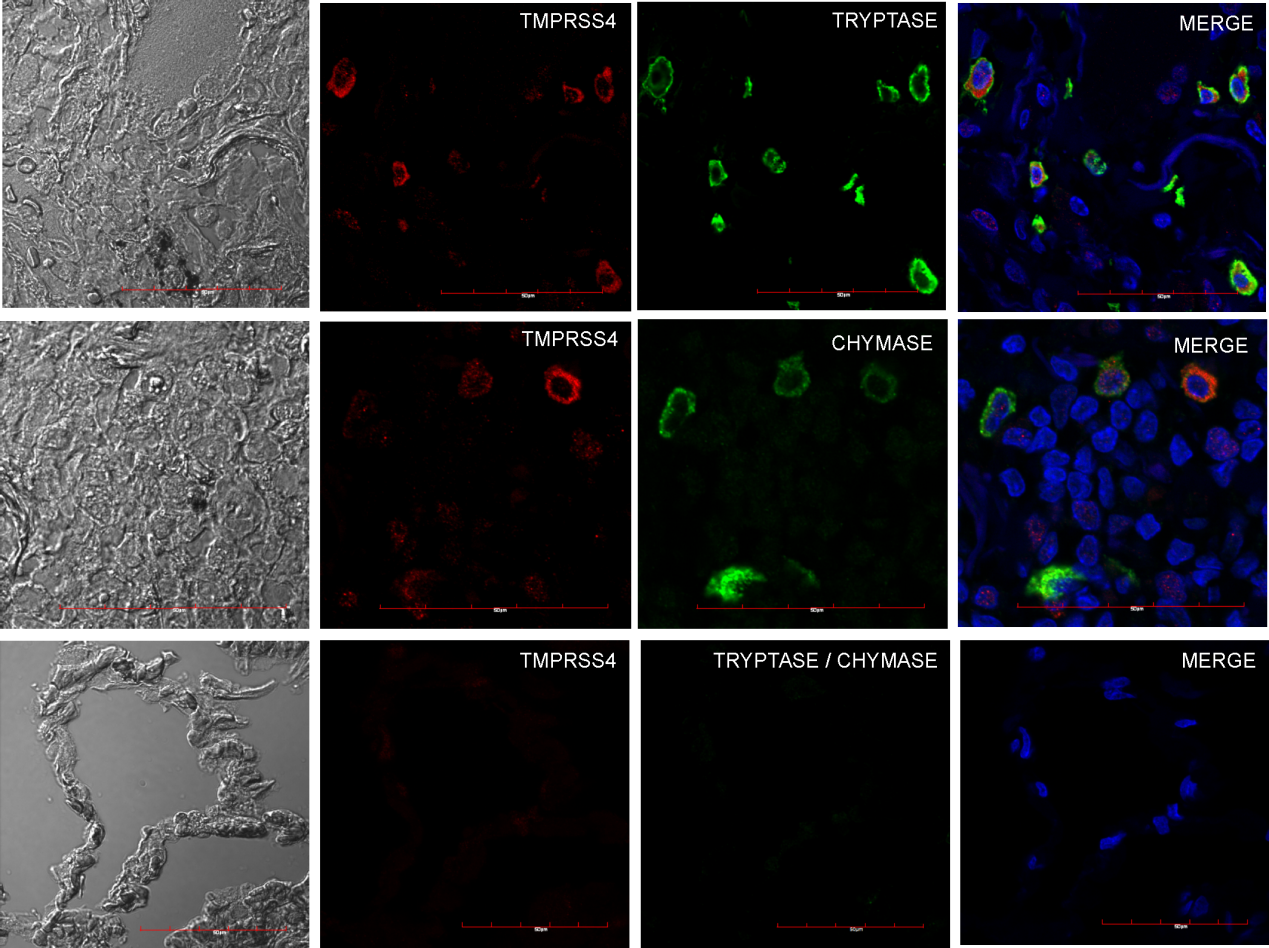
Co-localization of TMPRSS4, tryptase and chymase in IPF and control lungs. Representative images of immunofluorescence staining performed with specific antibodies against each molecule in lung tissue sections from IPF patients. Top and central row: IPF tissues coexpressing TMPRSS4 and tryptase or chymase; bottom row: normal lung showing no expression. Tissues were stained for TMPRSS4 (Dylight-549, red), tryptase (AF-488, green upper panel) and chymase (AF-647, green middle panel). The colocalization of TMPRSS4 and tryptase or chymase was determined by fluorescence microscopy and images were merged to resolve the co-localization of these proteins. Co-localization of TMPRSS4 was observed with both triptase and chymase. Data are representative of three independent experiments.

Sequential slides of a single IPF biopsy stained with TMPRSS4 specific antibody and toluidine blue suggested that the TMPRSS4-expressing interstitial cells could be mast cells and to confirm this finding, we co-localized TMPRSS4 with tryptase and chymase, two proteases present in the granular content of mast cells that are commonly used as markers of these cells. As illustrated in **Fig 3**, confocal microscopy analysis revealed the co-localization of TMPRSS4 with both enzymes in IPF samples.

### TMPRSS4 is expressed by epithelial and bronchial cells in vitro but not by fibroblasts

**Fig 4:**
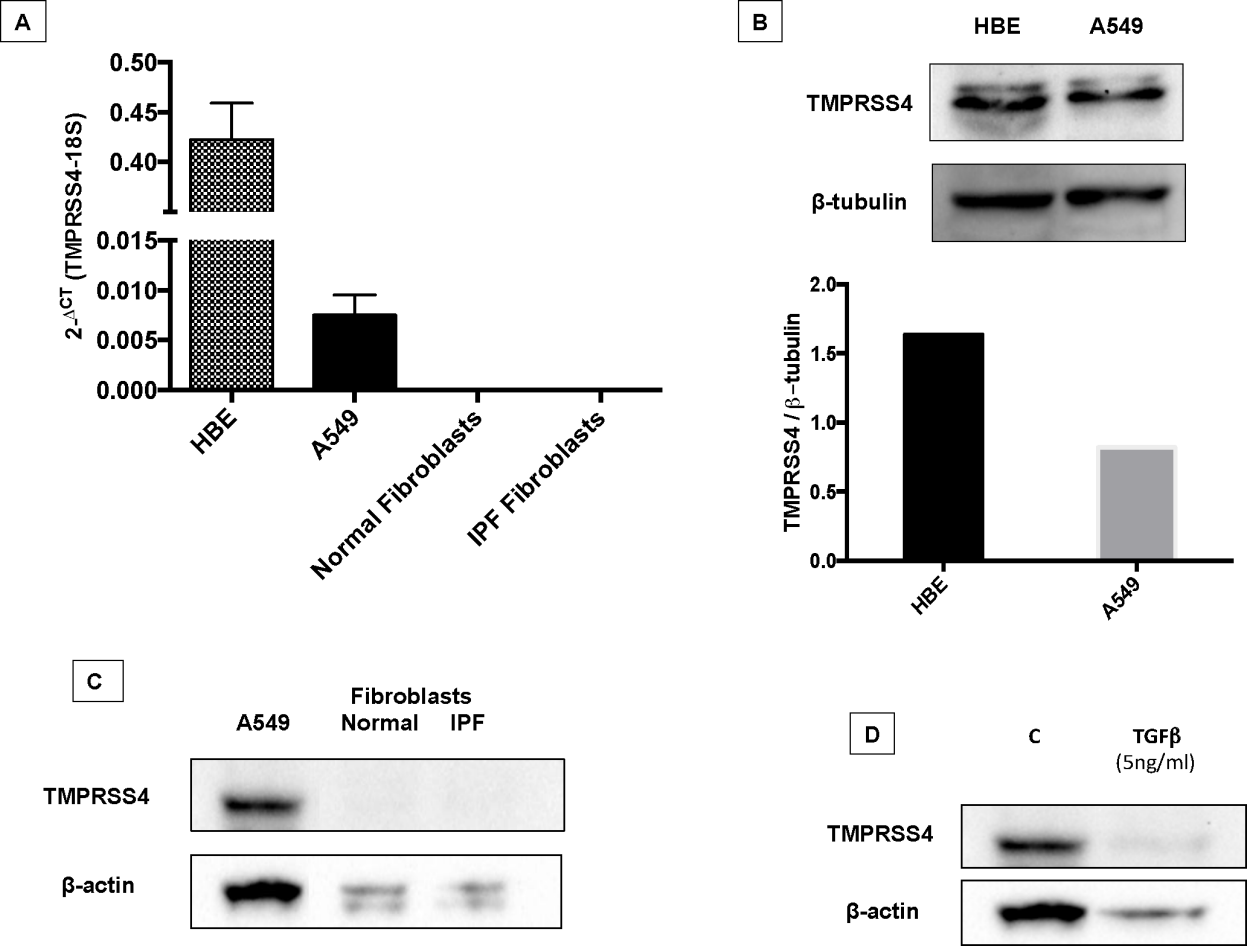
TMPRSS4 is expressed by alveolar and bronchial epithelial cells. Panel A: Gene levels of TMPRSS4 were quantified by real-time RT-PCR in A549 and HBE4-E6/E7 epithelial cells and in normal (n = 3) and IPF lung fibroblasts (n = 3). Expression was observed in both epithelial cell lines while no expression was found in fibroblasts. Panel B: Protein expression of TMPRSS4 (48 KDa) was confirmed by western blot in both epithelial cell lines. Panel C: Representative Western blot of TMPRSS4 in normal and IPF lung fibroblasts. A549 epithelial cells were used as positive control. Panel D: Western blot of A549 epithelial cells stimulated with TGFβ1 for 96 hours.

To corroborate the IHC results, we measured the gene and protein expression of TMPRSS4 in normal human and IPF lung fibroblasts and in two epithelial cell lines, one from alveolar origin (A549) and another of bronchial origin (HBE). As shown in **Fig 4A–4C**, neither normal nor IPF derived fibroblasts expressed TMPRRS4 while both epithelial cell lines did it at mRNA and protein level. We also explored the effect of TGF-β1 on the expression of TMPRSS4 in A549 epithelial cells, and a decrease of TMPRSS4 was observed at 96 hours (**Fig 4D**).

### TMPRSS4 increases the expression of E-cadherin

**Fig 5:**
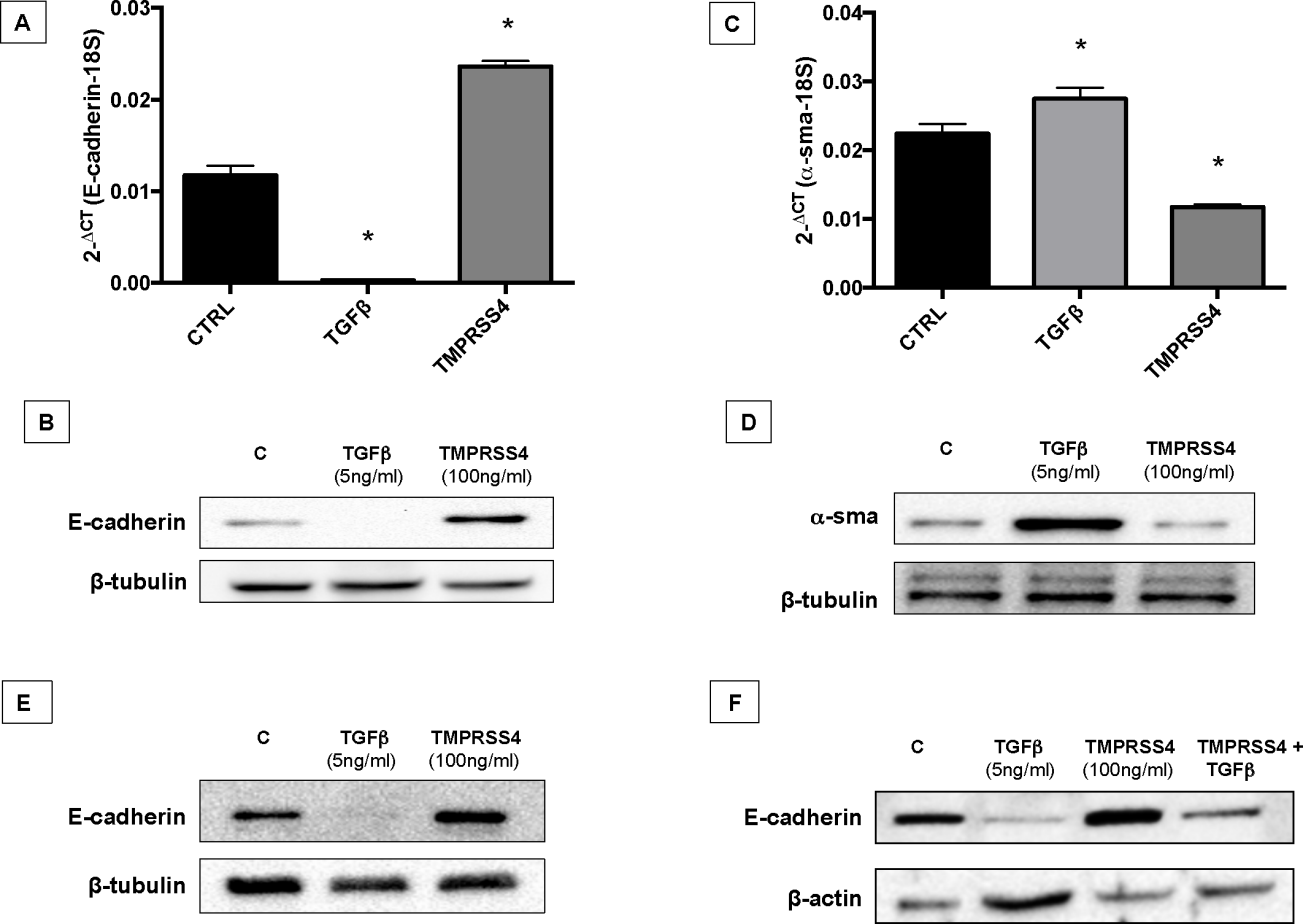
TMPRSS4 increases E-cadherin expression. Human alveolar epithelial cells (A549) were stimulated with TMPRSS4 (100 ng/mL) or TGF-β1 (5 ng/mL) and the expression of E-cadherin and α-SMA was determined by quantitative RT-PCR and Western blot. Panel A and C: At 4 days, TMPRSS4 significantly increased the level of E-cadherin mRNA and reduced α-SMA mRNA compared with control sample (*p <0.01). Data were normalized to the level of 18S rRNA. Panel B and D: Total protein from stimulated cells was extracted and western blot analysis performed with specific antibodies for E-cadherin and α-SMA. TGF-β1 was used as a positive control for EMT. Panel E: Rat (RLE-6TN) alveolar epithelial cells were stimulated with TMPRSS4 (100 ng/mL) or TGF-β1 (5ng/mL) for 4 days. Westerns are representative of three independent experiments. Panel F: Western blot analysis of control A549 epithelial cells and A549 stimulated with TGFβ1, TMPRSS4 or TGFβ1 plus TMPRSS4.

Prior evidence suggested that TMPRSS4 promotes EMT [**20**]. Since this process has been implicated in the pathogenesis of IPF [**21, 22**], we examined whether TMPRSS4 may induce EMT on alveolar epithelial cells. For this purpose the levels of E-cadherin and α-SMA were analyzed in TMPRSS4-treated and untreated epithelial cells. Unexpectedly, TMPRSS4 increased the expression of E-cadherin at the gene and protein levels (**Fig 5A and 5B**). Likewise TMPRSS4 reduced the expression of α-SMA gene by RT-PCR (**Fig 5C**), but gave variable results at the protein level where we observed either a decrease (**Fig 5D**) or no changes. The increase of E-cadherin induced byTMPRSS4 was confirmed in rat (RLE-6TN) alveolar epithelial cells (**Fig 5E**). Since the activation of TGF-β signaling causes epithelial to mesenchymal transition (EMT), a process that include the downregulation of the expression of E-cadherin, we thought to evaluate the impact of TMPRSS4 on this process. A549 epithelial cells were treated with TGF-β alone, TGF-β1 plus TMPRSS4, or TMPRSS4 alone, and the expression of the protein was examined at 4 days by Western blot. As shown in **Fig 5F**, TMPRSS4 partially inhibited the strong decrease of E-cadherin induced by TGF-β1, further supporting the effect of this type II transmembrane serine protease on the cell adhesion protein.

### TMPRSS4 reduces the growth rate of alveolar epithelial cells

**Fig 6:**
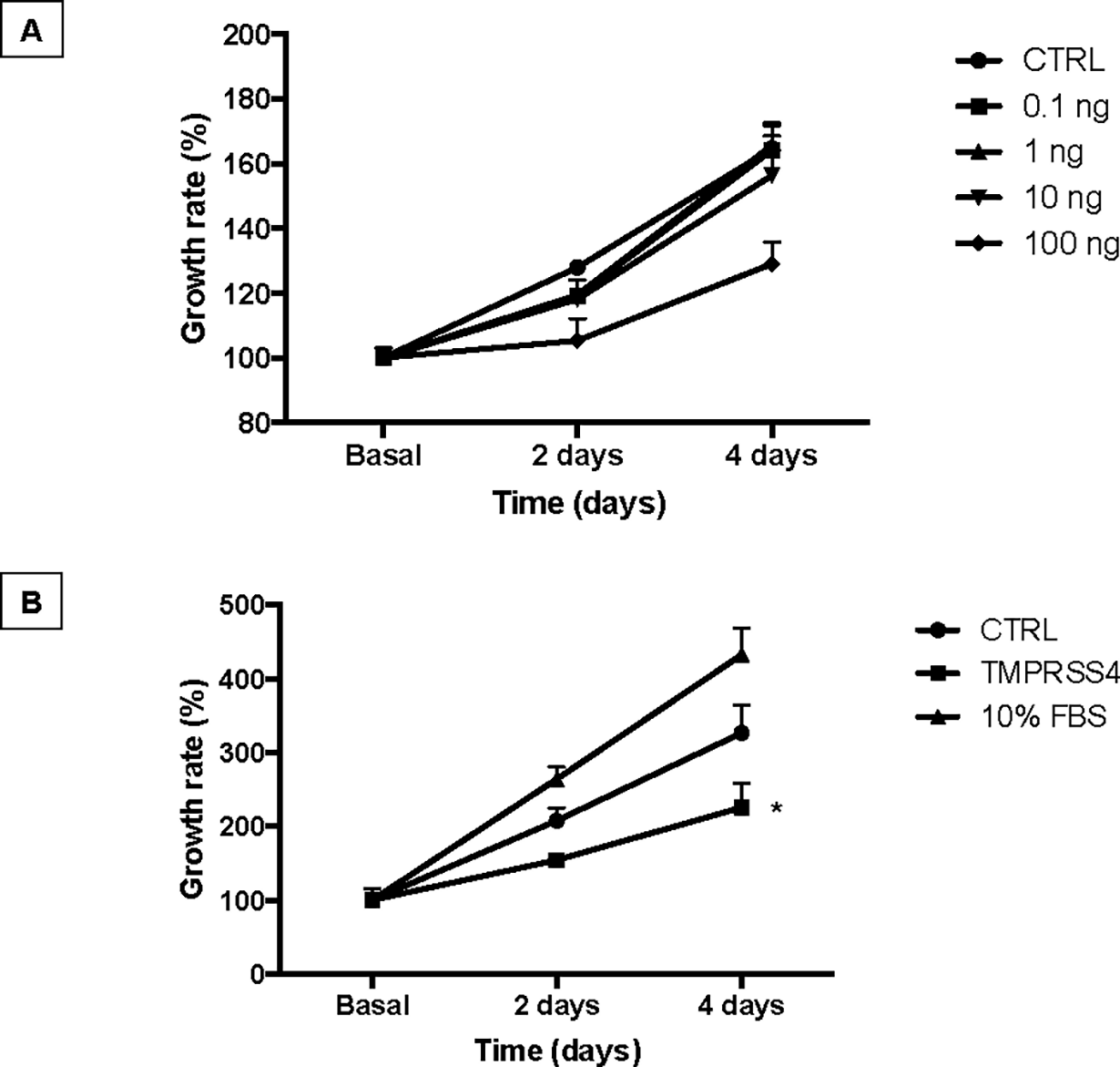
TMPRSS4 reduces alveolar epithelial growth rate. Panel A: Human alveolar epithelial cells (A549) were cultured in 48-well culture plates in medium without FBS and stimulated with TMPRSS4 (0.1, 1, 10 and 100 ng/mL). Panel B: The analysis was repeated with 100 ng/ml in two independent experiments. Cell numbers were estimated by WST-1 assay at 4 days of culture. Each point represents means ± SD of one representative experiment performed in triplicate. *P < 0.05.

Stimulation of A549 epithelial cell line with TMPRSS4 recombinant protein induced a decrease in the growth rate measured by cell viability WST-1 assay. The analysis of the time and dose-response curve showed that the inhibitory effect of TMPRSS4 depends on their concentration achieving a significant effect at a protein concentration of 100 ng/mL at 4 days after stimulation (**Fig 6A and 6B**).

### TMPRSS4 deficiency attenuates bleomycin-induced lung fibrosis in mice

**Fig 7:**
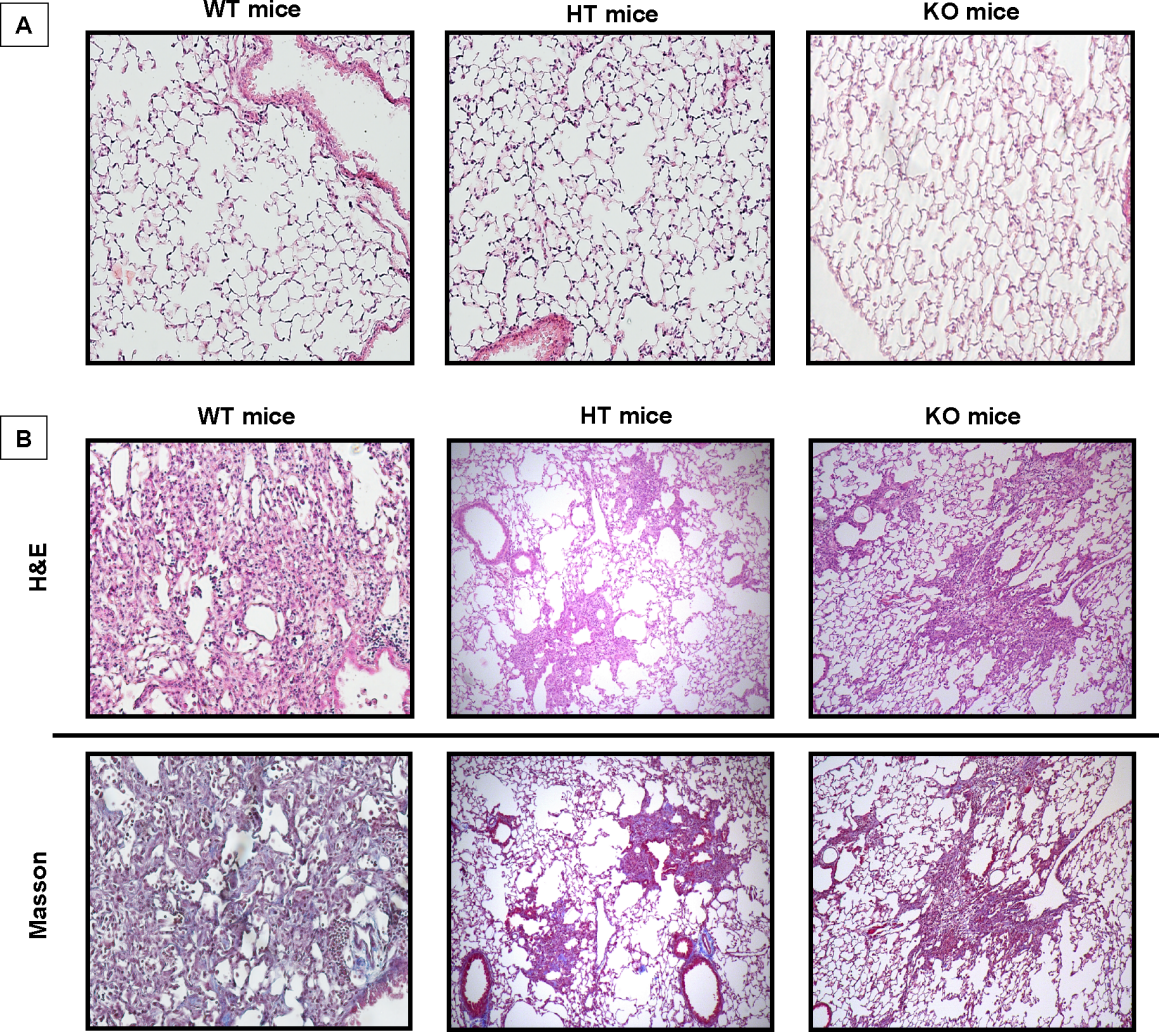
TMPRSS4 deficiency attenuates bleomycin-induced lung damage in mice. Wild type (WT), TMPRSS4 deficient (KO) and haplodeficient (HT) mice were instilled intratracheally with bleomycin (7 U/Kg) or saline solution and studied at 28 days. Panel A: control mice instilled with saline solution. Panel B: mice with bleomycin-induced pulmonary fibrosis. Histopathologic analysis was performed using hematoxylin and Masson trichrome staining.

**Fig 8:**
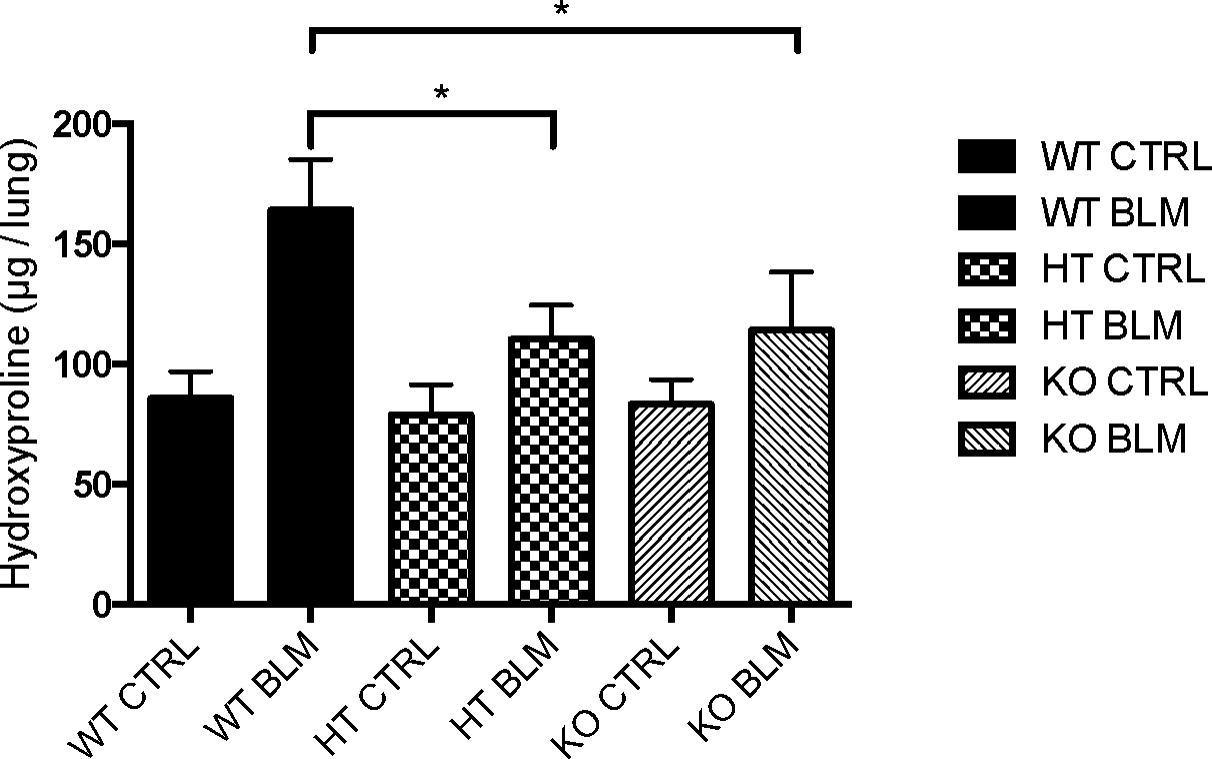
TMPRSS4 deficiency reduces bleomycin-induced collagen deposition in lungs. Wild type (WT), TMPRSS4 deficient (KO) and haplodeficient (HT) mice were instilled with Bleomycin (7 U/Kg) and studied at 28 days. Collagen content was quantified by hydroxyproline assay. Data are expressed as mean ± SD of 7–12 animals per group. *P< 0.01 compared with WT instilled animals.

To define the effect of TMPRSS4 on the development of lung fibrosis, wild-type and TMPRSS4 heterozygous (+/-) and deficient (-/-) mice were treated with a single instillation of bleomycin (7U/Kg) or saline solution and sacrificed at 28 days. The fibrotic response was examined by lung morphology and hydroxyproline content. Histopathologic assessment showed severe scarring in the wild type bleomycin-injured animals that was markedly reduced in TMPRSS4 knockout and haploinsufficient mice (**Fig 7**). The morphologic fibrotic index was significantly reduced in KO (560.7± 338.4) and HT (446.5 ± 259.7) mice versus WT animals (1100.0 ± 514.24) (p<0.01). We then determined collagen content by using the hydroxyproline assay on the whole left lung. On basal conditions, no differences in hydroxyproline content were found. At 28 days post-bleomycin instillation we observed a substantial increase of collagen concentration in the WT mice, which was significantly reduced in TMPRSS4 (+/-) and (-/-) deficient mice (164 ± 21.1 μg OH-Pro/lung versus 110.2 ± 14.3 μg OH-Pro/lung and 114.1 ± 24.2 μg OH-Pro/lung respectively; p<0.01) (**Fig 8**). No differences were observed between TMPRSS4 haploinsufficient and deficient mice.

**Fig 9:**
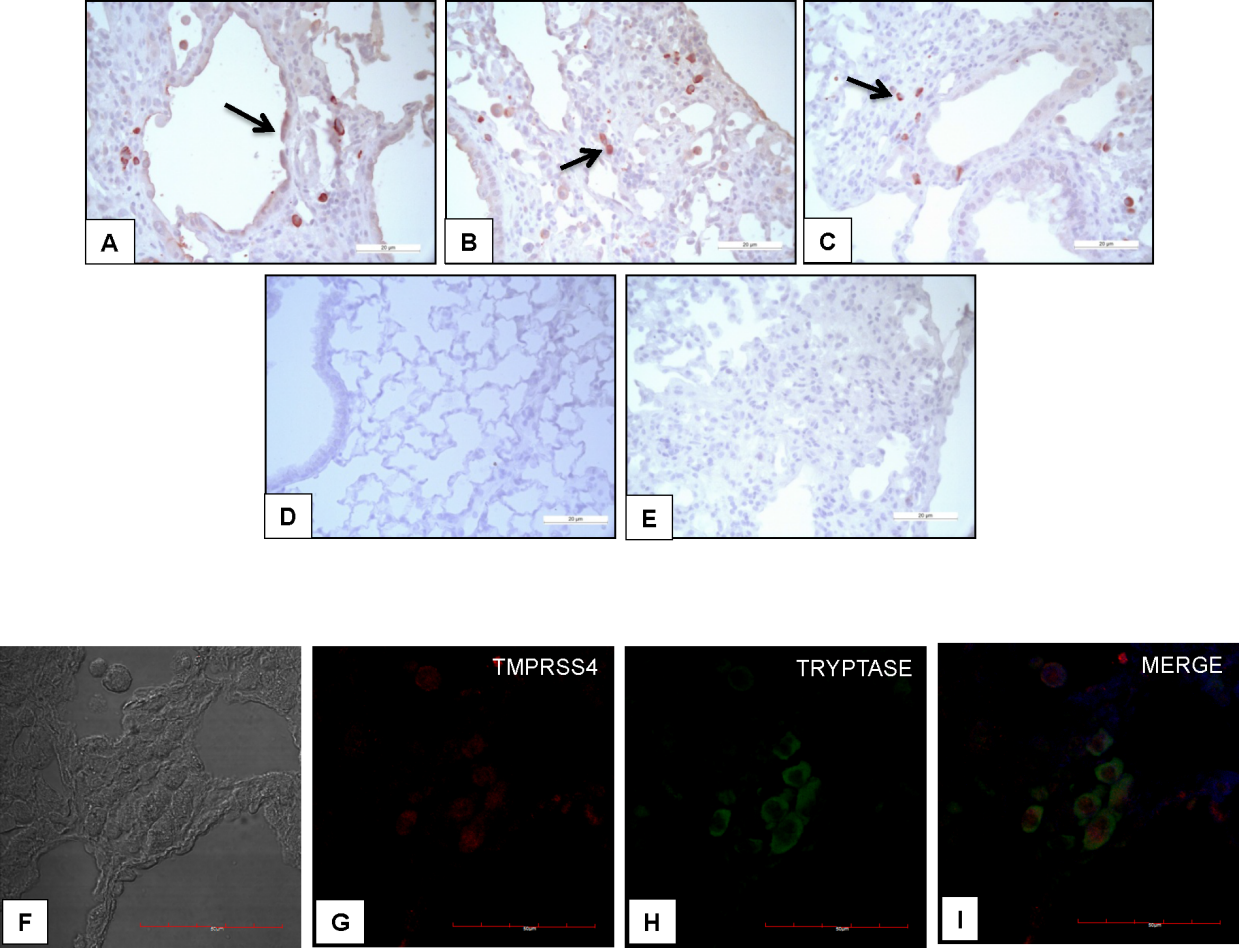
TMPRSS4 is expressed by epithelial and mast cells in bleomycin-induced pulmonary fibrosis. Representative photomicrographs of immunohistochemical staining performed with antibody against TMPRSS4 in lung tissue sections from WT mice injured with bleomycin. The immunoreactive enzyme was observed in epithelial (panel A, black arrow) and interstitial cells (panels B and C black arrows). Panel D: no positive staining was detected in normal lungs. Panel E: shows the negative control where the specific antibody was omitted. All sections were counterstained with hematoxylin. Panels F-I: Representative images of immunofluorescence staining performed with specific antibodies against TMPRSS4 and tryptase; Tissues were stained for TMPRSS4 (Dylight-549, red) and tryptase (AF-488, green). The colocalization of TMPRSS4 and tryptase was determined by fluorescence microscopy and images were merged to resolve the co-localization of these proteins.

As detected in IPF, in WT mice TMPRSS4 was expressed by epithelial cells and more intensely by interstitial cells (**Fig 9A–9E**). Immunocolocalization with tryptase demonstrated that the interstitial cells were mast cells (**Fig 8F–8I**).

## Discussion

IPF is a progressive and usually deadly disease of unknown etiology and uncertain pathogenesis. In the last years, a number of metalloproteases have been implicated in its pathogenesis not only through extracellular matrix remodeling but also in the abnormal behavior of lung cells [23–25]. However, studies with other proteases are scanty.

Type II transmembrane serine proteases comprises four subfamilies: (a) matriptase, (b) hepsin/transmembrane protease/serine (TMPRSS), (c) human airway trypsin-like (HAT)/differentially expressed in squamous cell carcinoma (DESC), and (d) corin [26].

Recently, it was revealed that the expression and activity of matriptase was up-regulated in IPF, affecting primarily the behavior of fibroblasts promoting their activation, proliferation, and migration [27]. Actually, the analysis of protein expression demonstrated a 2-fold increase of this enzyme in primary human pulmonary fibroblasts derived from IPF tissues compared with those from controls. The study also showed that inhibition of matriptase attenuated pulmonary fibrogenesis in vivo.

In our study we evaluated a member of another subfamily of the cell surface trypsin-like serine proteases, TMPRSS4, that in contrast to matriptase still remain orphan in the sense that no functions or substrates are currently known [28]. Most studies until know have focused on its implication in different cancers, where a growing body of evidence indicates that over-expression of TMPRSS4 among other effects enhances EMT-like process and induces migration [29] two events that has been described to occur in IPF [1,21, 22]. Studies in non-neoplastic diseases are scanty but it has been shown that TMPRSS4 may contribute to influenza virus replication and spread, and that a mutation of this protein causes a novel pediatric neurodegenerative disorder named autosomal recessive cerebral atrophy (ARCA) syndrome [30, 31].

To our knowledge, this is the first study dealing with this serine protease in any fibrotic disorder. We first explored the gene expression and putative localization of this molecule in IPF lungs and we found that TMPRSS4 was up-regulated and expressed mainly by mast cells and alveolar epithelial cells primarily in fibrotic areas of the lungs. In vitro, TMPRSS4 was expressed by bronchial and alveolar epithelial cells, and importantly, was absent in IPF and normal human lung fibroblasts, contrary to what was described with matriptase.

Surprisingly, stimulation of human alveolar epithelial cells with TMPRSS4 provoked a significant increase of the expression of E-cadherin, which was corroborated with epithelial cells obtained from rats. E-cadherin, the major cadherin in epithelial cells, is an important adhesion molecule that mediates tight binding to epithelial cells [32]. This molecule is critical to maintain the tissue structural integrity and among other functions, inhibits cell proliferation [33]. Supporting our data, growth rate was significantly decreased by stimulation with recombinant TMPRSS4. Our data also indicate that TMPRSS4 inhibits EMT induced by TGFβ1. EMT is a process that has been associated with the expansion of the fibroblast population and subsequent fibrosis. However, EMT is not always an all-or-none response but shows a spectrum of intermediary phases and may induce an intermediate phenotype, known as partial EMT state. Importantly, partial EMT can allow the efficient collective migration of cohesive epithelia maintaining their internal organization and in this way accelerate wound healing [34]. For example, partial EMT occurs in adult epidermal wound healing to facilitate the migration of keratinocytes during re-epithelialization accelerating wound closure. Likewise, the club cells in the bronchiole airways undergo a transient EMT program during the regeneration of the bronchiolar epithelium [35]. In this context, inhibiting partial EMT and consequently an appropriate epithelial cell migration, TMPRSS4 could contribute to abnormal fibrotic tissue remodeling after injury. We also observed that TGFβ1 seems to decrease the expression of TMPRSS4 suggesting a negative regulation by this mediator.

To determine whether TMPRSS4 is implicated in the development of experimental lung fibrosis, WT and TMPRSS4 (+/-) and (-/-) mice were challenged with bleomycin and the fibrotic response was measured by morphology and collagen content. Our data revealed an important role for TMPRSS4 in promoting bleomycin-induced pulmonary fibrosis since while a strong fibrosis was observed in the WT, a relatively modest increase in collagen deposition and preserved lung architecture was noticed in TMPRSS4 heterozygous and deficient mice after administration of bleomycin. Interestingly, we do not observe a gene-dose effect. There are several examples in the literature demonstrating that in some cases haploinsufficient mice behave very similar to completely deficient mice indicating a dominant effect of the haploinsufficiency [36, 37]. Similar to IPF, in the mouse model of lung fibrosis TMPRSS4 was also observed in epithelial cells and more strongly in mast cells.

The putative profibrotic mechanisms of TMPRSS4 however, are uncertain. Interestingly, strong signal was found in mast cells (MC) which are usually markedly increased in the fibrotic areas of lungs from patients with IPF [38]. Activated MCs, are usually found in close proximity to fibroblast foci and alveolar type II cells where they may release numerous mediators including a variety of proteases and cytokines that exert profibrotic activity [38]. Human lung mast cells have granules with distinct protease content, and have been classified as mast cells containing either tryptase only (MC_T_), chymase only (MC_C_) or both tryptase and chymase (MC_TC_) in their granules. These subtypes may change their phenotypes according modifications of the microenvironment [39]. In IPF, we have previously found a marked increase of TGF-β expression primarily in MC_TC_ located in small airways and alveolar parenchyma [40]. In the present study, we found by using confocal microscopy, that all these subtypes of mast cells express TMPRSS4 suggesting that this serine protease may be one of the profibrotic molecules expressed by these cells. However, how this enzyme modifies the behavior of mast cells is presently unknown. In addition, although weaker than in mast cells, TMPRSS4 was also found in lung epithelial cells that are critical for the development of IPF [1].

## Conclusions

Our results demonstrated by the first time that the cell surface trypsin-like serine protease TMPRSS4 is upregulated in IPF lungs and that the fibrotic response to bleomycin is attenuated in TMPRSS4-deficient mice suggesting a profibrotic role for this protease. However, further research is necessary to determine the mechanisms by which this enzyme contributes to enhance the lung fibrotic response.

## Supporting information

S1 Table Demographic and functional data of IPF and HP patients. (XLSX)
